# Review effects of radiation treatment on HPV-related vulvar cancer: a meta-analysis and systematic review

**DOI:** 10.3389/fonc.2024.1400047

**Published:** 2024-09-11

**Authors:** Wei Li, Lijun Zhai, Yinju Zhu, Fengjun Lou, Shiyu Liu, Ke Li, Liang Chen, Huankun Wang

**Affiliations:** Department of Radiation Oncology, The Third People’s Hospital of Dalian, Dalian Municipal Cancer Hospital, Affiliated Dalian Third People’s Hospital of Dalian Medical University, Dalian, Liaoning, China

**Keywords:** vulvar cancer, HPV, radiation therapy, sensitivity, outcome

## Abstract

**Objective:**

Vulvar carcinoma exhibits a robust correlation alongside HPV infection; however, the impact of HPV rank on the prognostic outcomes of radiation therapy within vulvar malignancies stays ambiguous. In the present study, we performed a comprehensive examination as well as meta-analysis to assess the influence of infection with HPV upon the long-term outlook as well as sensitivity of individuals with vulvar cancer undergoing radiation therapy.

**Methods:**

A meticulous examination of the existing research was conducted in accordance with the guidelines outlined in the Preferred Reporting Items for Systematic Reviews and Meta-Analyses (PRISMA) statement. A thorough search was conducted in the PubMed, Embase, Web of Science, as well as Cochrane Library databases, covering the entire available literature till April 1, 2023. The studies that met the inclusion criteria contained data about HPV infection and oncological outcomes in patients with vulvar cancer who received radiation therapy. This study was registered in PROSPERO (CRD42023417957).

**Results:**

We identified 12 retrospective studies meeting our inclusion criteria, which included a total of 3967 patients. Patients with HPV-associated vulvar cancer achieved a better overall survival rate after radiotherapy (HR=0.71, 95%CI: 0.54-0.93, P=0.01), and showed a significant improvement in disease-free survival (HR=0.75, 95%CI: 0.58-0.97, P=0.09) and progression-free survival (HR=0.31, 95%CI: 0.22-0.45, P,<0.01). Meanwhile, the complete remission rate after radiotherapy was higher for HPV-associated vulvar cancer patients (M-H=4.02, 95% CI: 1.87-8.61, P=0.0003), and the local control rate was better (HR=1.90, 95% CI: 1.15-3.15, P=0.01), exhibiting a reduced incidence of relapse within the field of study (HR=0.21, 95% CI: 0.10-0.42, P<0.001).

**Conclusion:**

In comparison to HPV-independent vulvar squamous cell carcinoma, patients with HPV-associated vulvar cancer exhibit higher sensitivity to radiotherapy, with a significant difference in prognosis. Further research should investigate the mechanisms underlying this high sensitivity to radiotherapy caused by HPV, and should be evaluated using high-quality randomized controlled trials.

## 1 Introduction

Vulvar tumors, a relatively uncommon malignancy, manifests in the vulvae and constitutes about five percent of carcinomas affecting the female sexual tract (1). This particular type of tumor predominantly emerges in women who have reached the after menopause stage (2). While cancer of the vulvar tract is a relatively uncommon occurrence, the incidence of it has exhibited an upward trend in the past few years, with a notable rise observed among the more youthful female population (3). Squamous cell carcinoma, a prevalent unhealthy variant, stands as one of the most frequently encountered form of vulvar tumors, probably developed from Lichen sclerosus (4, 5). As per the 2020 WHO classification guidelines for cancers of the female genital tract (6), vulvar carcinoma of squamous cells has the potential to be categorized into two distinct categories: HPV-associated or HPV-independent, based on its association with the presence or absence of human papillomavirus (HPV) contamination. The therapeutic strategy for cancer of the vulvar tract is primarily dictated by the histological composition of the tissue and the classification according to the International Federation of Obstetrics and Gynecology (FIGO) staging system (7). Early-stage cancer of the vulvar is commonly managed through a surgical procedure wherein the affected tissue is removed. The decision to employ adjuvant radiation therapy depends on various factors, including the condition of the surgical the profit margins. Individuals presenting with localized or advanced pathology may necessitate the administration of adjuvant radiotherapy in conjunction with surgical intervention, or alternatively, they might receive radiotherapy just like the primary therapeutic modality (8).

In the past 20 years, there have been significant breakthroughs in the field of image-guided radiotherapy (IGRT) and intensity-modulated radiotherapy (IMRT) technological advances have enabled radiotherapy to be delivered with greater precision and personalization, allowing for higher doses to be targeted to tumors while minimizing radiation exposure to healthy tissues (9, 10). In the context of vulvar cancer, these technological advancements have led to improved local control and reduced toxicity during radiotherapy (11). The AGO-CaRE-1 study (12) demonstrated that adjuvant radiotherapy in the inguinal region following surgery improves survival in individuals presenting via carcinoma of the vulvar along with the occurrence of dissemination in the inguinal lymphatic system. In this study, adjuvant radiotherapy for lymph node-positive patients showed improved survival outcomes. The impact of HPV status on radiotherapy outcomes in vulvar cancer, which is an important prognostic factor, remains an area of active investigation.

Persistent HPV infection is known to be a potential danger for gynecological and head and neck tumors (13). In the oropharynx, the presence of the infection determines better outcomes, due to radiosensitivity, so that de-escalation programs exists (14–16). In cervical cancer, squamous histology, determined in over 95% of cases by HPV, has better outcomes than other histologies (17, 18). This does not mean that in vulvar cancer the situation cannot be different. Several clinical studies have investigated the radiation sensitivity and prognosis of HPV-associated vulvar cancer patients, and older meta-analyses and analysis of prospective studies, have shown that the trend towards better outcomes is maintained (19–21), but a comprehensive meta-analysis on the impact of HPV infection on radiotherapy outcomes in vulvar cancer has not been published. Hence, our objective is to perform a comprehensive examination as well as meta-analysis of existing information pertaining to the radiation therapy forecasting of vulvar cancer related to HPV. Additionally, we target to evaluate the influence of infection with HPV upon the susceptibility of vulvar tumors to radiation treatment.

## 2 Methods

### 2.1 Criteria for admission and search methodology

This systematic review and meta-analysis included a comprehensive investigation of the Embase, PubMed, the Web of Science, along with the Cochrane Central Register of Controlled Trials archives to locate every pertinent study done in the context of English. published from inception until April 1, 2023. We used the following search terms: “HPV”, “human papillomavirus”, “vulvar cancer”, and “vulvar squamous cell carcinoma” to ensure that all relevant studies were included in our analysis.

Only studies that tested for HPV DNA or p16^INK4a^ and reported radiotherapy prognosis findings were incorporated within the framework of this comprehensive systematic review and meta-analysis. Furthermore, we conducted a thorough examination of the references mentioned in the encompassed papers in order to ascertain any further pertinent scientific literature. Two biologists (WL and LZ) conducted a thorough examination of the titles, summary sections, and complete documents pertaining to possibly suitable studies, adhering strictly to the established parameters for acceptance and rejection. To prevent any potential prejudice, they were unaware of the writers and journal identities associated with each study. Discrepancies were resolved through discussion with a third researcher (YZ). Only peer-reviewed articles published in English were considered, and preprints, meeting summaries or case reports were excluded. If data was missing from a study, we attempted to contact the corresponding authors through electronic mail in order to retrieve incomplete information. In the event that data that is absent cannot be acquired, the study was excluded from the analysis. The investigation was carried out in adherence to the PRISMA instructions and registered with the PROSPERO platform (registration number: CRD42023417957).

### 2.2 Data analysis

Two reviewers, FL and SL, autonomously retrieved the subsequent factors concerning each research: first author, publication year and journal, geographical region, sample size, age, FIGO stage, treatment method, HPV DNA or p16^INK4a^ detection data, radiotherapy prognosis data, and definition of each prognosis variable. After data extraction, the entire text and data will be reviewed by a third researcher (HW). The assessment of the quality of the research was conducted by two independent reviewers (KL and LC) in a manner consistent with the principles of the Newcastle-Ottawa Scale (NOS). The NOS evaluates the research studies according to three key classifications, namely the selection of cases, comparability of groups, and assessment of results. Studies alongside a star rating exceeding six were deemed to possess a commendable level of quality, whereas examines that garnered a star rating of five or six were regarded as possessing an average degree of quality. The meta-analysis exclusively incorporated research of high or moderate standard. After two rounds of debate failed to produce a unanimous decision, an additional investigator (HW) was brought in.

### 2.3 Inclusion criteria

The meta-analysis selection follows these inclusion criteria: (1) Study subjects: patients with vulvar cancer who underwent radiation therapy; (2) Study type: Clinical trials, as well as prospective and retrospective cohorts, along with case-control experiments; (3) Intervention measures: based on the expression of p16^INK4a^ or HPV DNA in vulvar cancer tissues (after surgical resection or biopsy sampling), patients were divided into a group of individuals who have tested positive for the HPV (experimental group) and a group lacking the presence of HPV (control group). Due to the strong correlation between p^16INK4a^ overexpression and HPV infection, investigations that documented p^16INK4a^ excessive expression were categorized as HPV positive. Subsequently, a subgroup evaluation was conducted based on this classification. Prognostic signs pertaining to radiation therapy encompass several key factors: the 5-year overall survival (OS), 5-year disease-free survival (DFS), 5-year progression-free survival (PFS), 5-year local control (LC), 5-year in-field relapse (IFR), and the rate of complete remission (CR) following treatment. These indicators serve as crucial measures in assessing the outcomes and efficacy of radiotherapy interventions. There exist no limitations pertaining to the minimum patient count required for the inclusion of each study within the analysis. In instances where multiple articles pertaining to analogous individuals and employing identical detection methodologies within a shared cohort are encountered, solely the most extensive or contemporary investigations shall be incorporated.

### 2.4 Exclusion criteria

(1) Original research with non-rigorous experimental design or incorrect statistical methods (such as incomplete data, unclear outcome evaluation indicators, or unreasonable design); (2) Medical case reports, reviews, preprints, and conference abstracts.

### 2.5 Statistical analysis

Review Manager 5.4.1 (Nordic Cochrane Centre, The Cochrane Collaboration) was employed to carry out the meta-analysis. The assessment of inter-study disparity was conducted by employing the Cochran chi-square (χ2) examination. If the value of I^2^ is greater than 50%, it signifies a substantial level of heterogeneity. In the case where I^2^ falls between 20% and 50%, it shows an average amount of heterogeneity. Conversely, if I2 is less than 20%, it implies a low level of heterogeneity. The analysis of studies exhibiting significant heterogeneity was conducted using the random-effects model, while studies with mild and minimal heterogeneity were examined using fixed-effects models. The utilization of a forest plot was employed to visually display the outcomes of individual investigations as well as the collective findings. The Mantel-Haenszel (M-H) model was employed to assess the association between two categorical variables, and the Hazard Ratio (HR) along with 95% confidence intervals (CIs) was utilized for the prognostic analysis. To elucidate the origins of heterogeneity, a meticulous sensitivity analysis was performed by systematically excluding individual inclusion studies, particularly in cases where the results exhibited substantial heterogeneity. The assessment of publication bias was conducted by employing the Begg funnel plot and Egger’s test. Engage Digitizer 12.1 (Download address: https://markummitchell.github.io/engauge-digitizer/) was employed to extract survival data in cases where it was not readily accessible within the referenced studies (22, 23).

## 3 Results

### 3.1 Study inclusion and features

**Figure 1:**
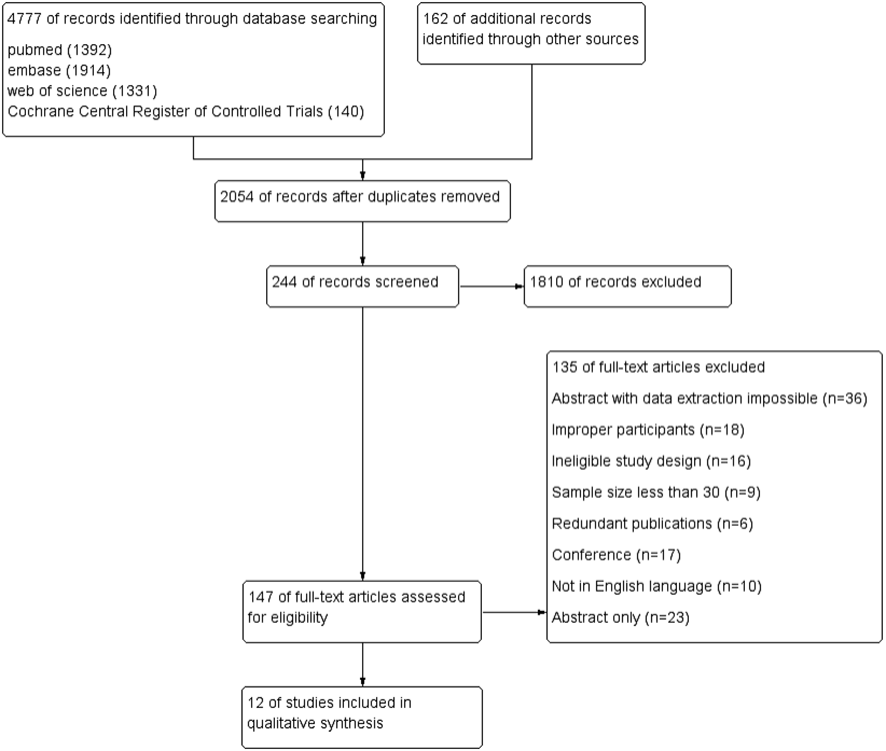
Procedure for reporting systematic reviews and meta-analyses according to the preferred criteria.

The meta-analysis included 12 eligible studies, as depicted in **Figure 1**. These studies involved 3967 patients from various countries, with 39–1247 individuals included in each research. Out of these, 8 studies reported HPV status based on FIGO staging, while 9 studies provided data on 5-year overall survival (OS), 6 studies focused on radical surgical treatment and reported 5-year disease-free survival (DFS), and 4 studies provided data on 5-year progression-free survival (PFS). Furthermore, 3 studies presented 5-year in-field relapse (IFR) data, while 2 studies each provided 5-year local control (LC) and complete remission (CR) rates after radiotherapy. Of the 12 studies, 4 identified HPV status via p16INK4a immunohistochemistry, while the rest of the studies used HPV DNA to determine HPV infection. The geographical distribution of the studies included 5 in North America, 5 in Europe, and 2 in the Asia-Pacific region. For more detailed information, please refer to **Table 1**, which summarizes the primary characteristics of these studies.

**Table 1:** Primary features of research that constitute the basis of the comprehensive review.

Author (year)	Country	Patients	HPV+	HPV-	Age (Years)	Treatment	Follow-up (Months)	Outcome
Woelber (2022) (24)	Germany	360	75	87	68.9	(1),(5)	17.2	(2),(3)
Horne (2018) (25)	USA	73	33	40	73	(2),	13.4	(2),(3),(5),(6)
Proctor (2019) (26)	Canada	48	26	22	67	(3),(5)		(1),(2),(5),(6)
Lee (2016) (27)	USA	57	15	41	75	(1),(2),(3),(4),(5)	58.0	(1),(2),(4),(6)
Arians (2019) (28)	Germany	75	13	62	68	(1),(2),(3),(4)	28.3	(1),(2),(3)
Yap (2018) (29)	Canada	40	14	26	69.5	(1),(2),(3),(4)	58.8	(1),(2),(4),(6)
Sznurkowski (2016) (30)	Poland	85	37	48	68	(1)	89.2	(1)
Kim (2015) (31)	Korea	56	15	20	71	(3),(4)	33.6	(1),(2)
Alonso (2011) (32)	Spain	98	19	79	68	(2),(5)	45.6	(1),(2),(6)
Barlow (2020) (33)	Australia	117	63	54	71	(1),(3),(4)	72.0	(2),(6)
Dohopolski (2019) (34)	USA	39	10	29	71	(1)	25.7	(1),(2),(4),(6)
Kortekaas (2020) (35)	Netherland	413	75	338	73	(1)	30.0	(1),(2),(6)

Treatment: (1).Adjuvant radiotherapy; (2).Neoadjuvant radiotherapy; (3).Radiotherapy alone; (4).Concurrent chemoradiotherapy; (5).Salvage radiotherapy.

Outcome: (1).5-year OS; (2).5-year DFS; (3).5-year LC; (4).5-year IFR; (5).CR rate; (6).FIGO stage.

### 3.2 5-year overall survival

**Figure 2:**
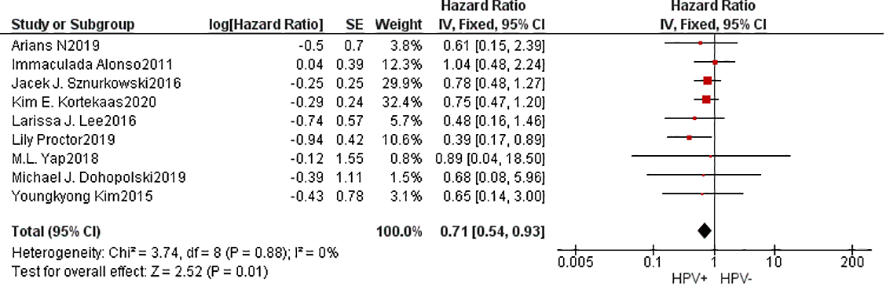
Forest plot of comparison: 5-year OS in HPV positive group is better.

A cumulative of 9 investigations were encompassed within the present meta-analysis. The findings of the heterogeneity examination indicated that I²=0%, P=0.88, suggesting no substantial heterogeneity observed. Consequently, it can be inferred that the studies were comparable in nature. Utilizing a fixed effects model, the observed outcomes: HR=0.71, 95%CI: 0.54-0.93, P=0.01. These findings indicated that individuals belonging to the HPV positive group exhibited superior 5-year OS compared to those in the HPV negative group subsequent to undergoing radiation treatment with statistical significance. To explore potential sources of heterogeneity, subgroup analysis was performed based on the method used to determine HPV positivity (p16^INK4a^ or HPV DNA) and the geographical source of the study(**Supplementary Material**). However, the results of subgroup analysis did not show significant differences, indicating that these factors did not affect the overall results. Furthermore, the Begg funnel plot and Egger test indicated no evidence of significant publication bias (**Figure 2**).

### 3.3 Survival without sickness and progression for five years

Information concerning 6 separate studies investigating the 5-year DFS were incorporated into the comprehensive meta-analysis. The findings of the heterogeneity examination indicated that I²=15%, P=0.32, suggesting a low substantial heterogeneity observed, and the observed outcomes: HR=0.81, 95%CI: 0.64-1.03, P=0.09. After removing the data one by one, it was found that heterogeneity decreased(I²=0%, P=0.59) when the study of Alonso was removed, and the result is HR=0.75, 95%CI: 0.58-0.97, P=0.09. These findings indicated that individuals belonging to the HPV positive group exhibited superior 5-year DFS compared to those in the HPV negative group subsequent to undergoing radiation treatment with statistical significance.

**Figure 3:**
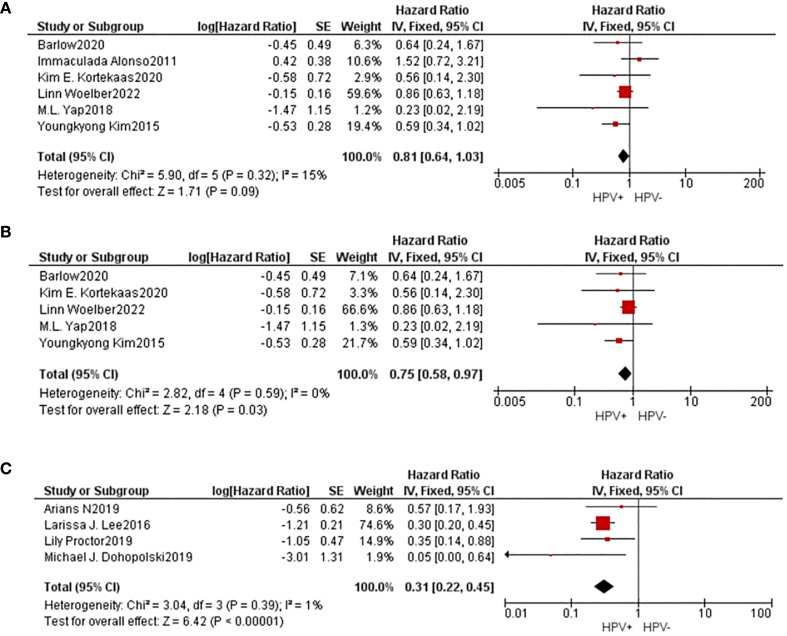
A comparative forest diagram: Statistical analysis of the forest plot shows that the HPV positive and negative groups do not vary significantly in 5-year DFS **(A)**. However, if the study of Alonso was removed, heterogeneity will be eliminated, and showed that 5-year DFS **(B)** and 5-year PFS **(C)** in HPV positive group is better.

4 studies provided data on 5-year PFS, and the same results occurred(I²=1%, P=0.39, HR=0.31, 95%CI: 0.22-0.45, P<0.01). The Begg funnel plot and Egger test were employed to identify any potential publication bias, and no notable asymmetry was detected in the funnel plot (**Figure 3**).

### 3.4 Radiotherapy sensitivity

**Figure 4:**
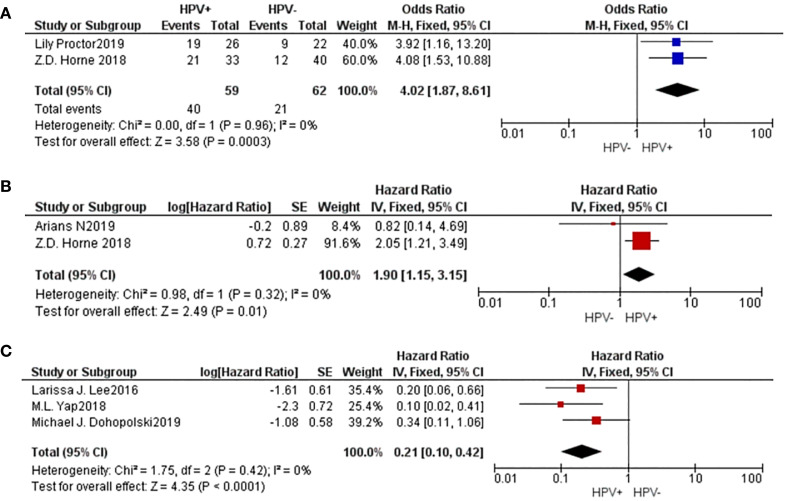
A comparative forest plot: The HPV positive group had a worse CR rate **(A)** and 5-year LC **(B)**, and a higher 5-year IFR **(C)**.

2 studies were included to evaluate the complete response (CR) rate of radiotherapy. The findings revealed that the population with a positive HPV status exhibited a higher rate of complete response (CR) compared to the group with a negative HPV status (M-H=4.02, 95% CI: 1.87-8.61, P=0.0003). Additionally, 2 studies were included to evaluate the 5-year local control (LC) rate after radiotherapy, and the results showed that the HPV positive group had better 5-year LC after radiotherapy (HR=1.90, 95% CI: 1.15-3.15, P=0.01). Moreover, 3 investigations yielded information regarding the 5-year in-field relapse (IFR), revealing that the cohort beneficial for HPV exhibited a lower 5-year IFR subsequent to radiation treatment (HR=0.21, 95% CI: 0.10-0.42, P<0.001). These results suggested that HPV-positive vulvar cancers have a higher radiosensitivity (**Figure 4**).

### 3.5 FIGO stage

**Figure 5:**
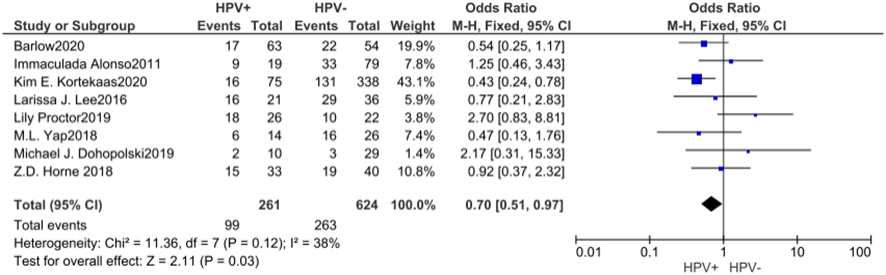
Forest plot of comparison: The proportion of patients reaching stages III and IV was lower in the HPV positive.

A cumulative of 8 investigations were encompassed within the present meta-analysis, as well as the outcomes of the heterogeneity test revealed an acceptable amount of heterogeneity across the diverse studies, with an I² value of 38% and a P-value of 0.12, suggesting a degree of comparison. Utilizing a fixed effects model, the amalgamated effect size was determined to be M-H=0.70, with a 95% confidence interval ranging from 0.51 to 0.97, and a corresponding p-value of 0.03. This observation indicates that the prevalence of individuals progressing to stages III and IV was comparatively lower among those who tested positive for HPV, and this disparity exhibited statistical significance. According to the analysis of the Begg funnel plot as well as Egger test, there was no apparent presence of publication bias (**Figure 5**).

## 4 Discussion

The treatment of vulvar cancer is based on individualization, with surgery being the primary treatment for early-stage cases, and individualized radical surgery can provide significant survival benefits (20, 36, 37). However, for locally advanced or advanced patients, extensive surgical intervention may result in poor postoperative quality of life, psychological acceptance, and treatment efficacy (38). As a result, patients may require adjuvant or neoadjuvant radiotherapy and chemotherapy before or after surgery, or radiotherapy as the primary treatment modality (39). Thus, understanding the prognostic factors of radiotherapy patients is crucial for clinical doctors. Among the many risk factors for vulvar cancer, HPV infection is crucial (40), and the radiation sensitivity of HPV-positive patients is a subject of interest. Despite evidence of higher radiosensitivity and better prognosis in HPV-induced tumors associated with head and neck cancer (41–43),, HPV18 positivity has been shown to be an independent risk factor in gynecologic tumors, such as cervical cancer (44–46). After radiotherapy, the patients with persistent positive HPV had poor prognosis and poor sensitivity of radiotherapy (44, 47). Consequently, recent studies, such as AGO-CaRE-1 study (24), have focused on the impact of HPV status on radiotherapy prognosis and sensitivity in vulvar cancer. As far as we are aware, this meta-analysis represents the first effort to assess the predictive significance of HPV condition in radiation-treated patients suffering from vulvar cancer.

The HPV is a type of virus characterized by its double-stranded, circular DNA structure. It exhibits a particular propensity for infecting the skin and mucosal tissues of human beings. The presence of this specific variable has been established as a recognized risk for the formation of cutaneous and mucosal abnormalities, specifically observed in the vulvar, cervical, vaginal, perianal, and oral regions. Furthermore, this factor has the potential to induce the formation of different types of precancerous lesions as well as tumors (48). Specifically, the presence of persistent high-risk HPV infection poses a notable risk for the onset of vulvar cancer, particularly among young women (49). Recent scientific investigations have substantiated the prevailing notion that the worldwide prevalence of HPV infection among individuals afflicted with vulvar cancer has escalated to an alarming 40% (50). Anti-HPV vaccination has shown that it can reduce related tumors by over 90%, so that even cervical cancer, the most frequent, will become a rare disease, as per the WHO program (51). However, for unvaccinated older generations and people in poor areas, it is a topic of interest.

In this comprehensive examination and synthesis of data, it was observed that individuals diagnosed with vulvar carcinoma who exhibited HPV infection displayed a more favorable 5-year overall survival rate subsequent to radiation therapy if compared with the HPV negative cohort. This conclusion is supported by the amalgamated hazard ratio. Additionally, the included studies showed a relatively low positive rate of HPV in locally advanced and advanced patients, indicating a lower probability of lymph node positivity in HPV-positive patients. The HPV positive rate ranged from 17.33% to 54.17%, which is consistent with the overall HPV infection rate for vulvar cancer of around 40% (50). These findings suggest that HPV positive patients may have a lower degree of tumor malignancy and overall better survival benefits, which is in line with previous research conclusions (52).

What’s more, our meta-analysis showed a significant advantagement in 5-year DFS and PFS after radiotherapy between the HPV-positive and -negative groups. And we observed that the CR rate and 5-year LC were higher in the HPV positive group, while the 5-year IFR was lower. These findings suggest that vulvar cancer patients with HPV positivity exhibit better radiation sensitivity, resulting in better treatment outcomes after radiotherapy. This result was consistent with the clinical findings of Woelber et al. (24). Studies from different countries and authors strongly suggested that HPV-positive status may lead to better radiotherapy outcomes.

The mechanism by which HPV affects radiation sensitivity in vulvar cancer is not well understood. However, research on cervical cancer, another gynecological tumor, has shed some light on this topic. Recent studies have found that HPV positive patients with cervical cancer exhibit different radiation sensitivity depending on the HPV subtype (53). In the context of HPV, it has been observed that individuals who test positive for HPV16 may exhibit a heightened sensitivity to radiation therapy. Conversely, those who are infected with HPV18 or multiple strains of HPV may display a reduced responsiveness to such treatment (44). Some studies suggested that the prognosis of these patients is poor (46), but other authors considered that the prognosis of patients with multiple infections is still better than that of HPV-negative patients (45). After HPV infection occurs, E6 is the major oncogene that integrates into the host chromosome, transcribes and translates to form the E6 oncoprotein, which leads to the generation of tumor cells by inactivating the tumor suppressor pathway of p53 and PRB. The E6 protein binds to the intracellular e6-associated protein (E6AP), which alters the molecular configuration of E6 to form the E6/E6AP/p53 trimer complex, leading to p53 degradation, uncontrolled cell division, and affecting cell repair and apoptosis (54, 55). On the other hand, radiotherapy can induce DNA double-strand breaks and induce the phosphorylation and stabilization of p53 directly or indirectly. p53 protein can activate DNA repair proteins and inhibit cell growth cycle, it made the cell growth cycle stay on the node of s and G1 phase, which led to the enhancement of the sensitivity of radiotherapy (56). Based on the known mechanisms of HPV-induced tumorigenesis, it is possible that HPV infection in vulvar cancer may also lead to increased radiation sensitivity and better prognosis, as seen in cervical cancer.

However, this study still has several limitations that need to be addressed. The statistical support for the function of HPV infection in the prognosis of vulvar cancer treated with radiation may be lacking due to a lack of study on the overall survival rate of vulvar carcinoma and, much more so, on the susceptibility and future prospects of vulvar cancer radiation therapy. Additionally, there is a lack of standardization in determining HPV status. In different studies, the detection methods used to determine HPV positivity vary, with two detection methods available: p16^INK4a^ immunohistochemistry and HPV DNA. Although subgroup analysis did not show any differences between these two detection methods, previous studies have confirmed that p16^INK4a^ does not fully represent HPV positivity (57), which may lead to inaccurate results. Thirdly, current studies generally fail to analyze HPV typing in patients. And based on the experience of cervical cancer, different types of HPV infection may lead to completely different treatment outcomes. Forthly, the treatment methods of each patient vary due to their various clinical and histological characteristics, which cannot be ignored. In different regions, differences in medical resources may make it difficult to determine the differences in treatment prognosis. Finally, due to the lack of basic experimental support, we have made reasonable extrapolations through relevant studies on cervical cancer, which is also a gynecological tumor, regarding the better radiotherapy prognosis of HPV-positive patients. However, it is still worth discussing whether this speculation is consistent with the facts. This study’s findings need confirmation by larger-scale studies with more data, as well as by the most recent cytological along with molecular assays.

## 5 Conclusion

The outcomes of the meta-analysis suggest that HPV-positive vulvar cancer patients have better radiation sensitivity and radiotherapy prognosis compared to HPV-negative patients. The HPV status can be a useful predictive indicator for the radiotherapy prognosis in vulvar cancer patients and can assist while making decisions on patient care.

## Author contributions

WL: Writing – original draft, Writing – review & editing. LZ: Writing – original draft, Writing – review & editing. YZ: Writing – original draft, Writing – review & editing. FL: Writing – original draft, Writing – review & editing. SL: Writing – original draft, Writing – review & editing. KL: Writing – original draft, Writing – review & editing. LC: Writing – original draft, Writing – review & editing. HW: Writing – original draft, Writing – review & editing.
