# Supplementary material for: Review effects of radiation treatment on HPV-related vulvar cancer: a meta-analysis and systematic review
Source: Front Oncol. 2024 Sep 11;14:1400047. doi: 10.3389/fonc.2024.1400047 (PMC11422069; doi:10.3389/fonc.2024.1400047)
Supplement: Supplementary file 1 [file Image1.pdf]

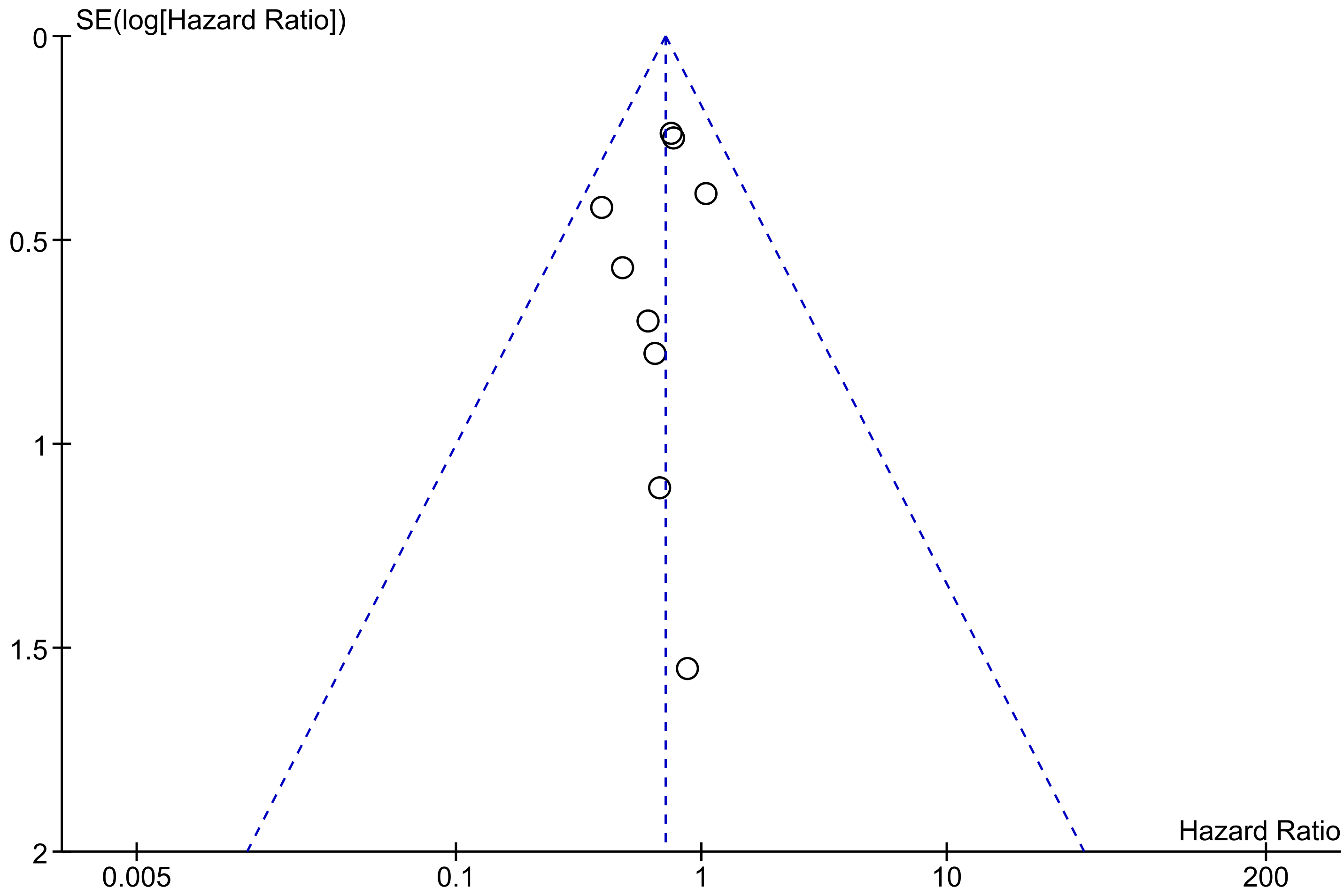

The Begg funnel plot and Egger test of 5-year OS

Study or Subgroup	log[Hazard Ratio]	SE	Weight	Hazard Ratio IV, Fixed, 95% CI
**2.1.1 p16**				
Lily Proctor2019	-1.24	0.49	8.0%	0.29 [0.11, 0.76]
Michael J. Dohopolski2019	-0.39	1.11	1.6%	0.68 [0.08, 5.96]
**Subtotal (95% CI)**			**9.6%**	**0.33 [0.14, 0.80]**
Heterogeneity: Chi² = 0.49, df = 1 (P = 0.48); I² = 0%				
Test for overall effect: Z = 2.46 (P = 0.01)				
**2.1.2 HPV DNA**				
Arians N2019	-0.5	0.7	3.9%	0.61 [0.15, 2.39]
Immaculada Alonso2011	0.04	0.39	12.6%	1.04 [0.48, 2.24]
Jacek J. Sznurkowski2016	-0.25	0.25	30.7%	0.78 [0.48, 1.27]
Kim E. Kortekaas2020	-0.29	0.24	33.3%	0.75 [0.47, 1.20]
Larissa J. Lee2016	-0.74	0.57	5.9%	0.48 [0.16, 1.46]
M.L. Yap2018	-0.12	1.55	0.8%	0.89 [0.04, 18.50]
Youngkyong Kim2015	-0.43	0.78	3.2%	0.65 [0.14, 3.00]
**Subtotal (95% CI)**			**90.4%**	**0.76 [0.57, 1.01]**
Heterogeneity: Chi² = 1.48, df = 6 (P = 0.96); I² = 0%				
Test for overall effect: Z = 1.87 (P = 0.06)				
**Total (95% CI)**			**100.0%**	**0.70 [0.54, 0.92]**
Heterogeneity: Chi² = 5.07, df = 8 (P = 0.75); I² = 0%				
Test for overall effect: Z = 2.54 (P = 0.01)				
Test for subgroup differences: Chi² = 3.09, df = 1 (P = 0.08), I² = 67.7%				

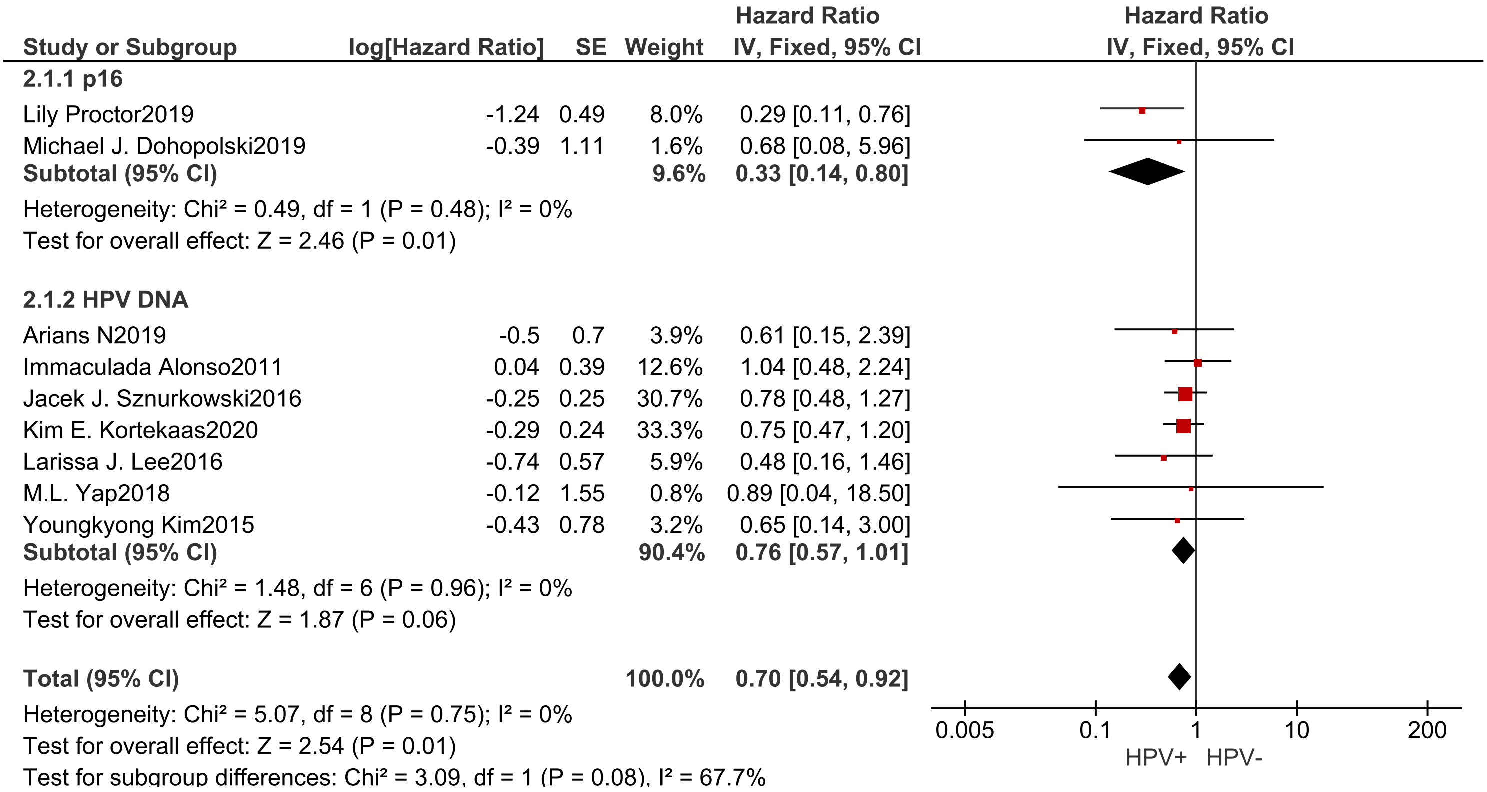

The subgroup analysis of 5-year OS (p16 vs HPV DNA), the results did not show significant differences

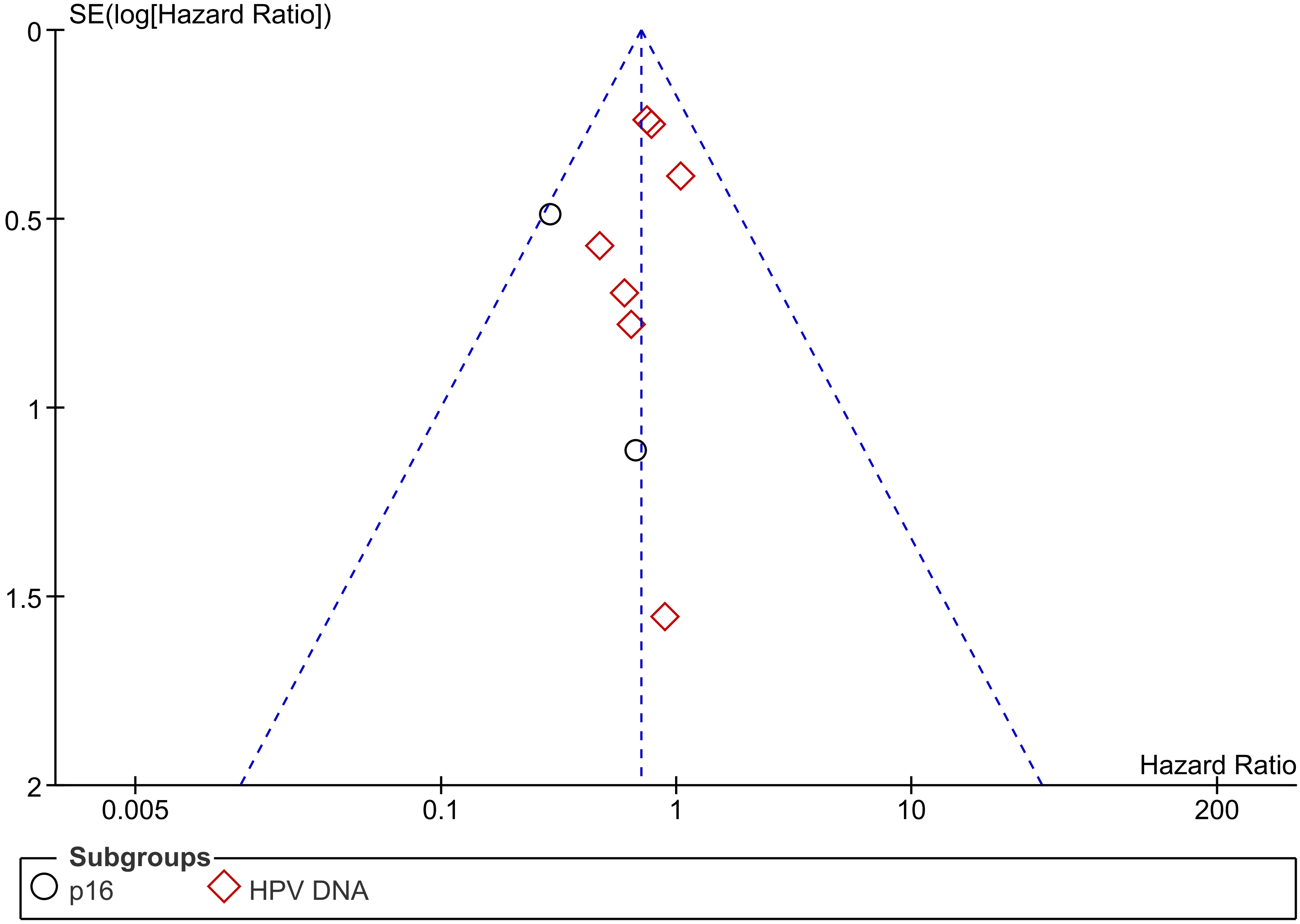
SE(log[Hazard Ratio])
0
0.5
1
1.5
2
Hazard Ratio
0.005
0.1
1
10
200
Subgroups
p16
HPV DNA

Study or Subgroup	log[Hazard Ratio]	SE	Weight	Hazard Ratio IV, Fixed, 95% CI	Hazard Ratio IV, Fixed, 95% CI
**2.2.1 North America**					
Larissa J. Lee2016	-0.74	0.57	5.9%	0.48 [0.16, 1.46]	
Lily Proctor2019	-1.24	0.49	8.0%	0.29 [0.11, 0.76]	
M.L. Yap2018	-0.12	1.55	0.8%	0.89 [0.04, 18.50]	
**Subtotal (95% CI)**			**14.7%**	**0.38 [0.19, 0.76]**	
Heterogeneity: Chi² = 0.77, df = 2 (P = 0.68); I² = 0%					
Test for overall effect: Z = 2.71 (P = 0.007)					
**2.2.2 Europe**					
Arians N2019	-0.5	0.7	3.9%	0.61 [0.15, 2.39]	
Immaculada Alonso2011	0.04	0.39	12.6%	1.04 [0.48, 2.24]	
Jacek J. Sznurkowski2016	-0.25	0.25	30.7%	0.78 [0.48, 1.27]	
Kim E. Kortekaas2020	-0.29	0.24	33.3%	0.75 [0.47, 1.20]	
**Subtotal (95% CI)**			**80.6%**	**0.79 [0.59, 1.07]**	
Heterogeneity: Chi² = 0.70, df = 3 (P = 0.87); I² = 0%					
Test for overall effect: Z = 1.51 (P = 0.13)					
**2.2.3 Asia & Pacific**					
Michael J. Dohopolski2019	-0.39	1.11	1.6%	0.68 [0.08, 5.96]	
Youngkyong Kim2015	-0.43	0.78	3.2%	0.65 [0.14, 3.00]	
**Subtotal (95% CI)**			**4.7%**	**0.66 [0.19, 2.30]**	
Heterogeneity: Chi² = 0.00, df = 1 (P = 0.98); I² = 0%					
Test for overall effect: Z = 0.65 (P = 0.51)					
**Total (95% CI)**			**100.0%**	**0.70 [0.54, 0.92]**	
Heterogeneity: Chi² = 5.07, df = 8 (P = 0.75); I² = 0%					
Test for overall effect: Z = 2.54 (P = 0.01)					
Test for subgroup differences: Chi² = 3.61, df = 2 (P = 0.16), I² = 44.5%					

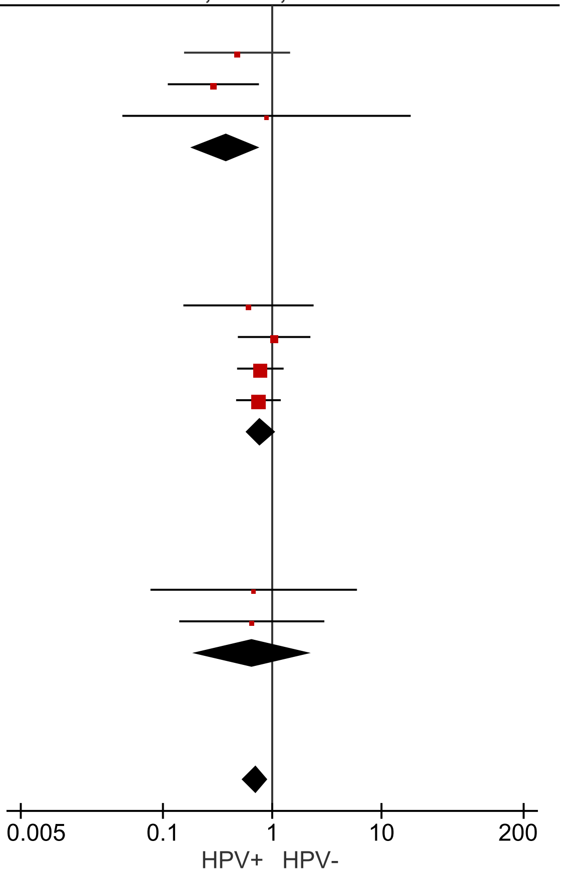

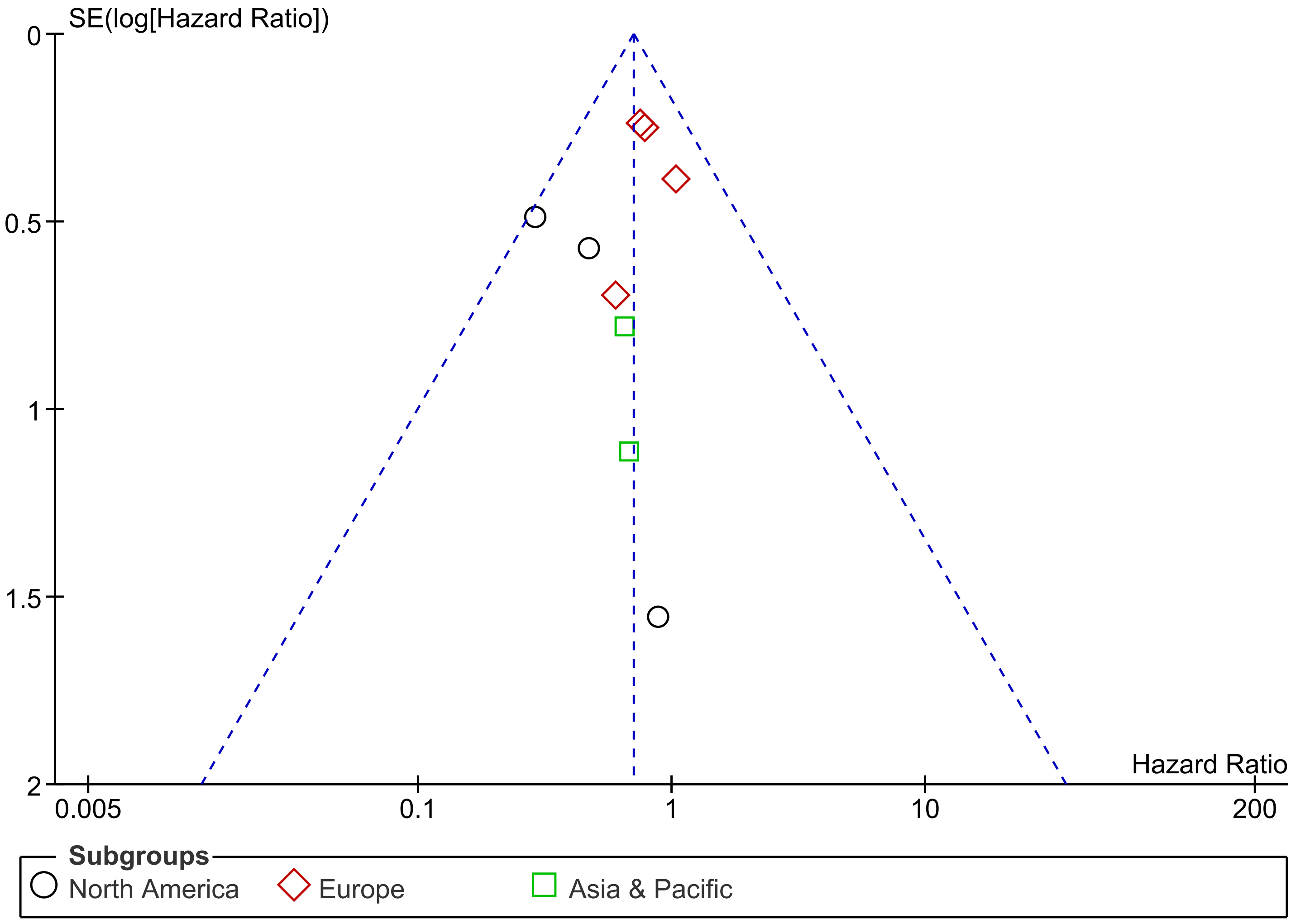
SE(log[Hazard Ratio])
0
0.5
1
1.5
2
Hazard Ratio
0.005
0.1
1
10
200
Subgroups
North America
Europe
Asia & Pacific

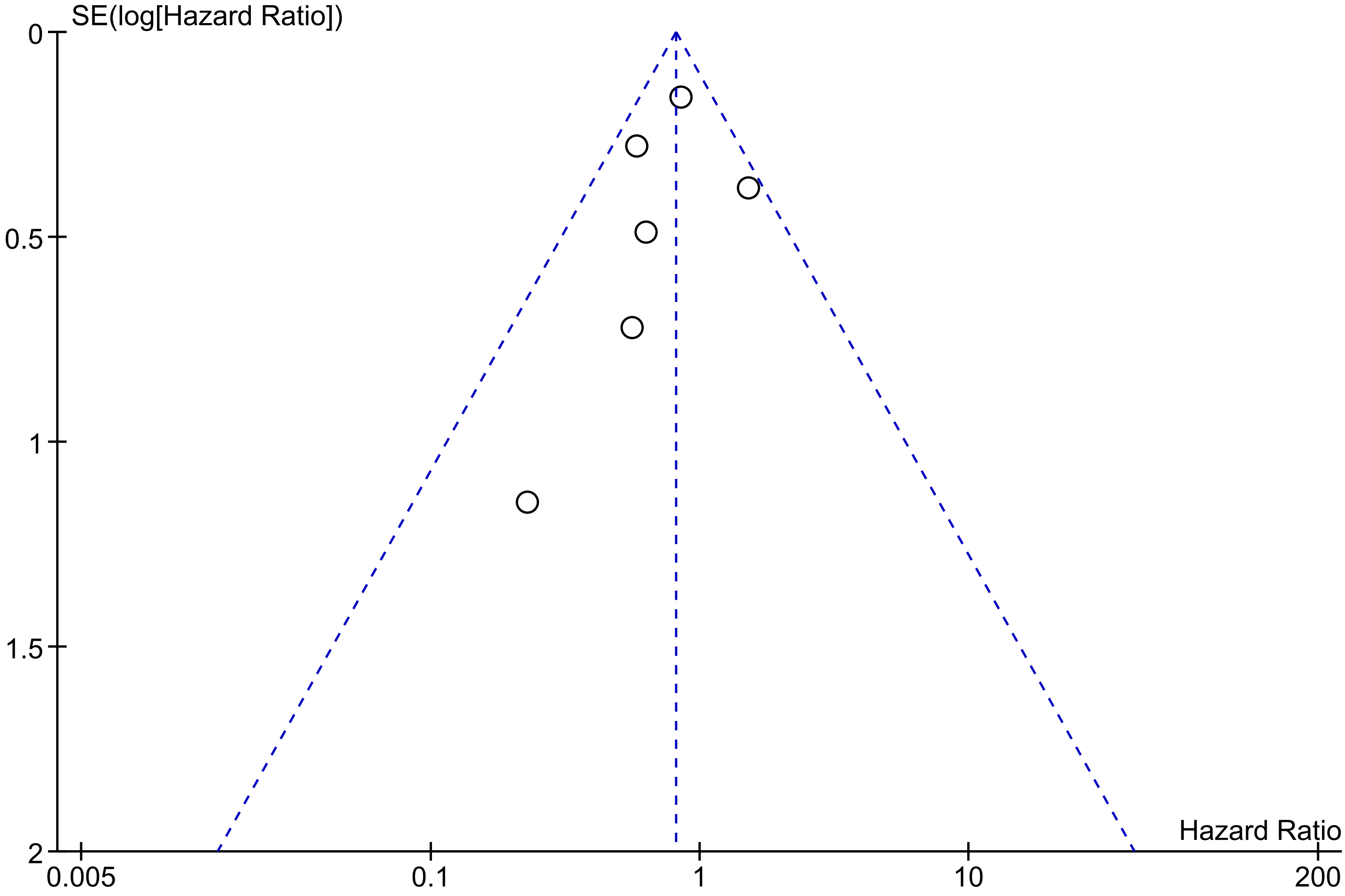

The Begg funnel plot and Egger test of 5-year DFS

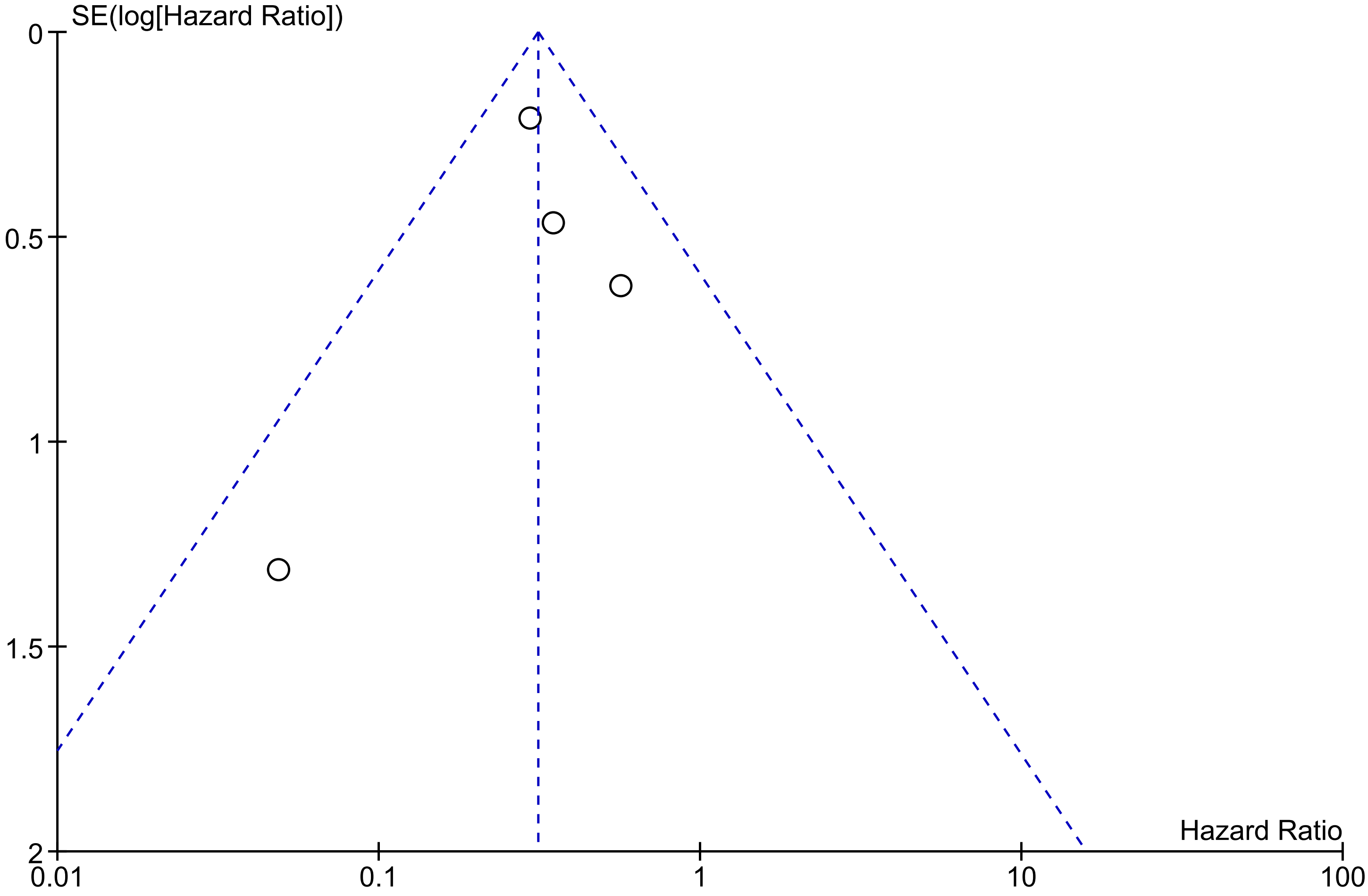

The Begg funnel plot and Egger test of 5-year PFS

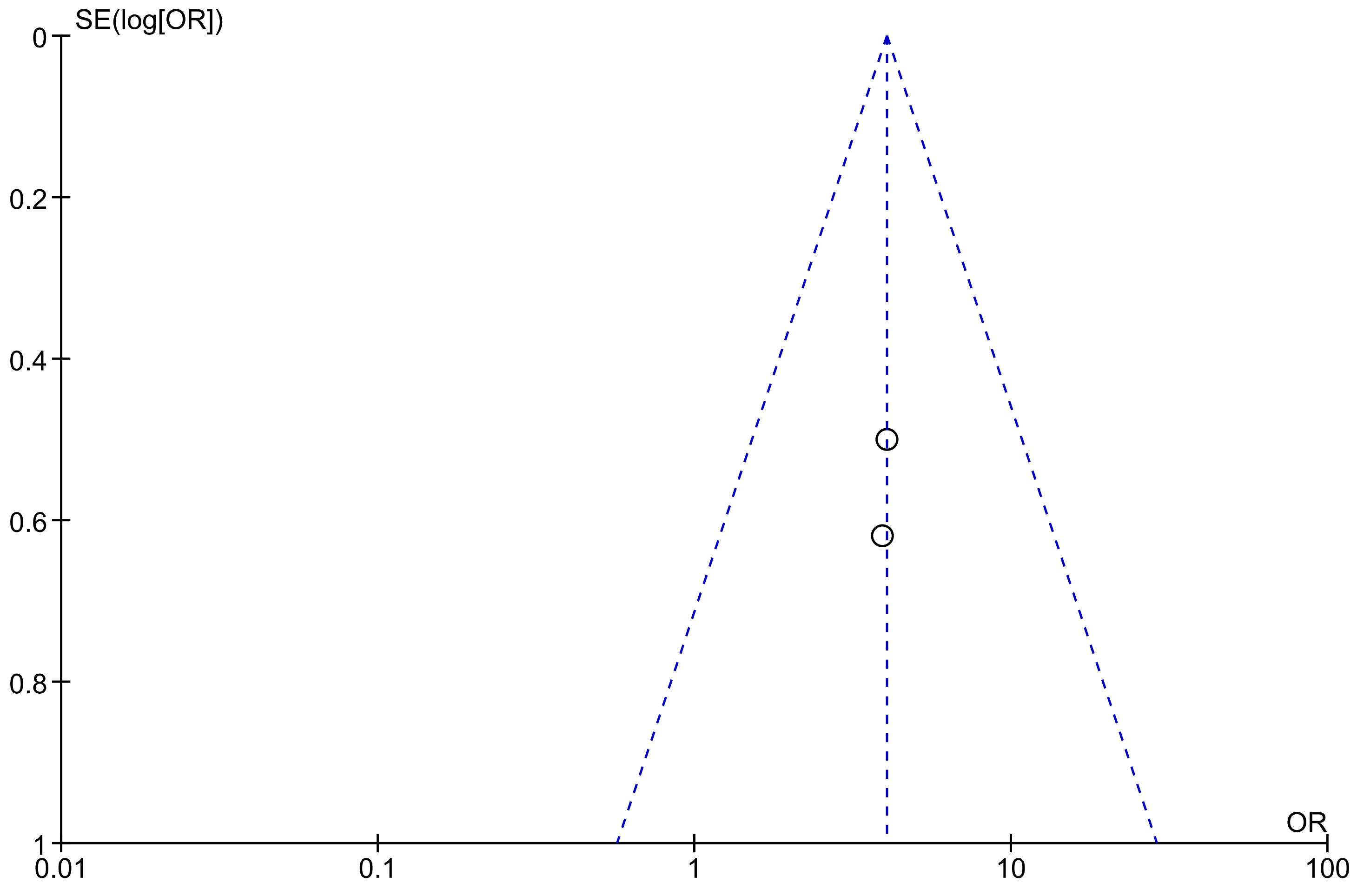

The Begg funnel plot and Egger test of CR rate

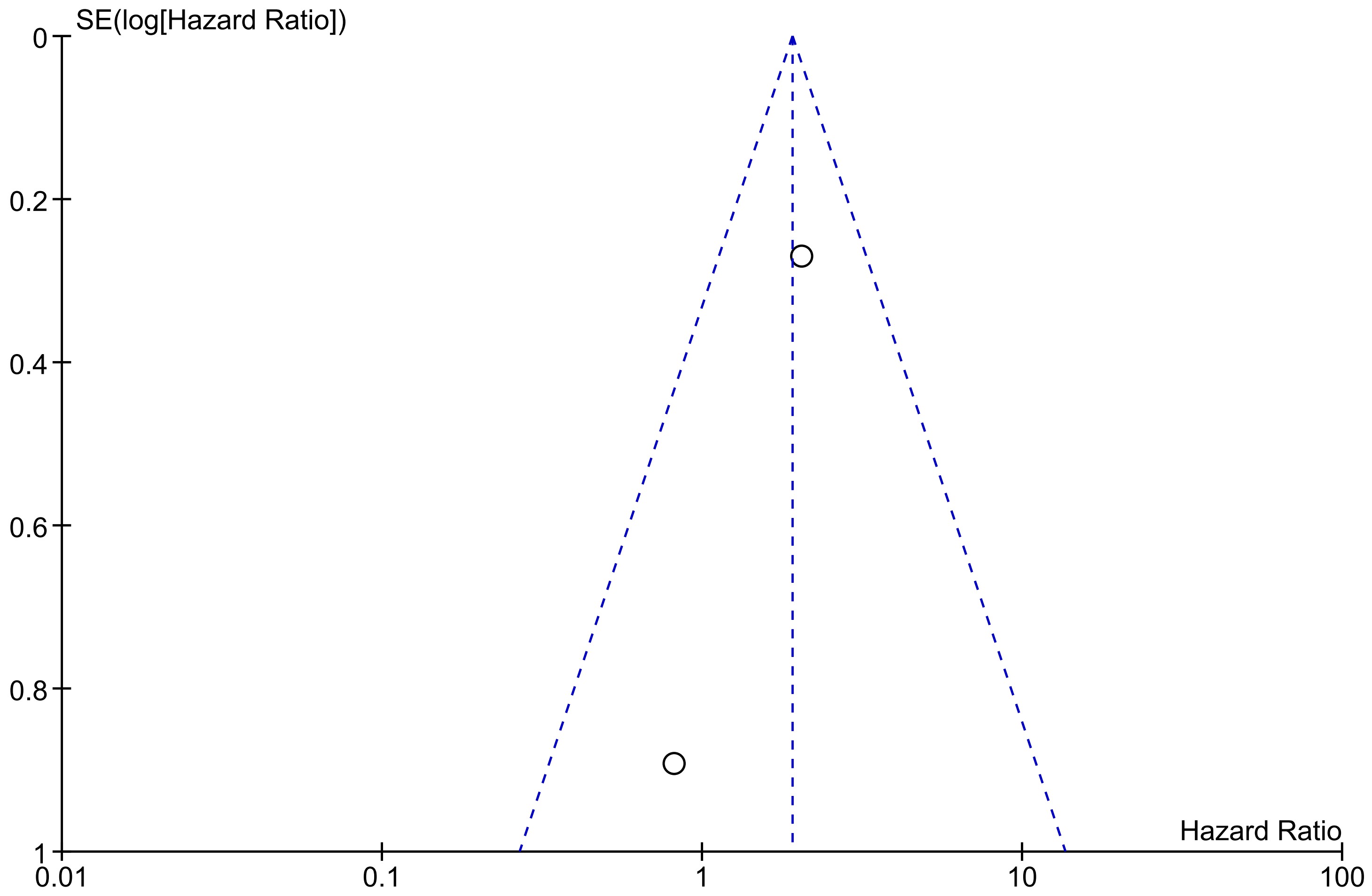

The Begg funnel plot and Egger test of 5-year LC

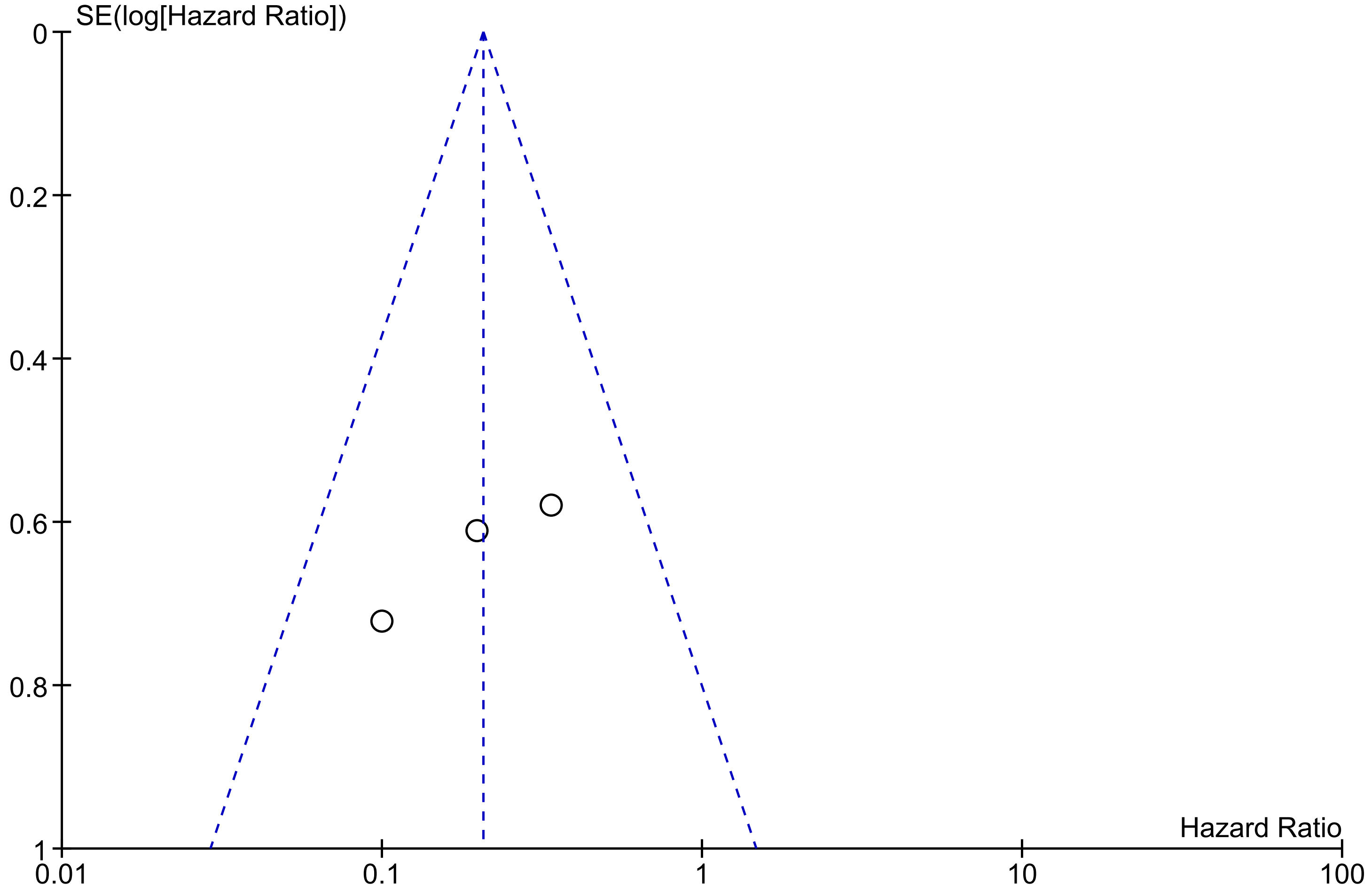

The Begg funnel plot and Egger test of 5-year IFR

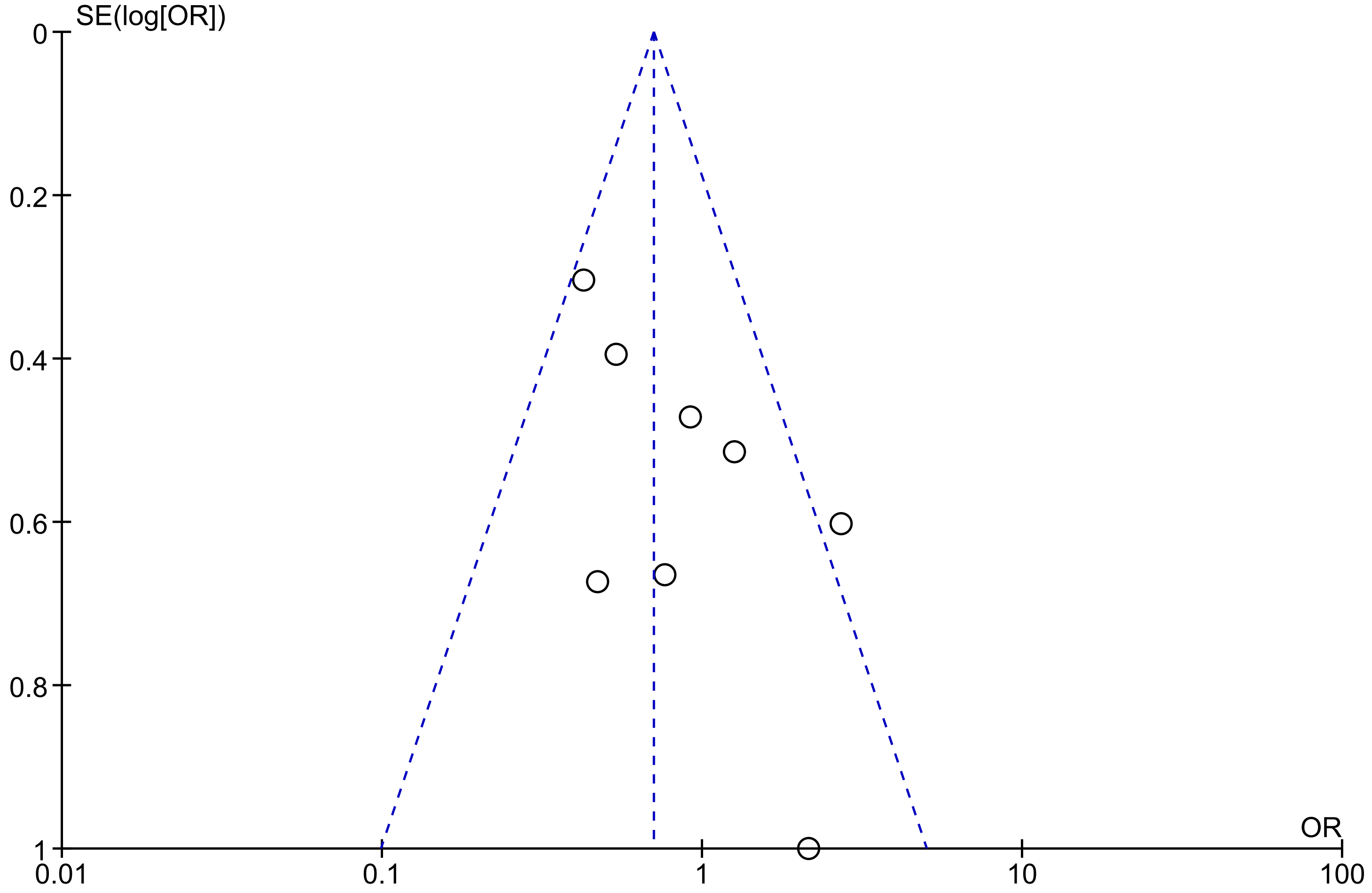

The Begg funnel plot and Egger test of FIGO stage

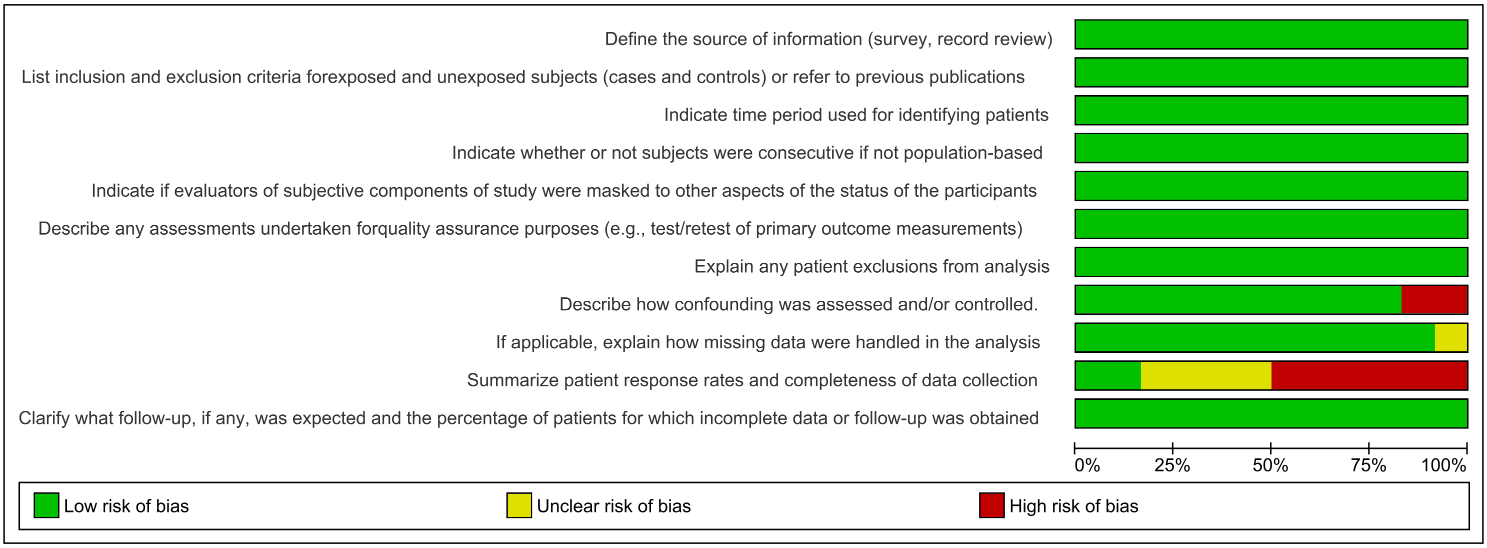

Risk of bias graph

	Define the source of information (survey, record review)	List inclusion and exclusion criteria forexposed and unexposed subjects (cases and controls) or refer to previous publications	Indicate time period used for identifying patients	Indicate whether or not subjects were consecutive if not population-based	Indicate if evaluators of subjective components of study were masked to other aspects of the status of the participants	Describe any assessments undertaken forquality assurance purposes (e.g., test/retest of primary outcome measurements)	Explain any patient exclusions from analysis	Describe how confounding was assessed and/or controlled.	If applicable, explain how missing data were handled in the analysis	Summarize patient response rates and completeness of data collection	Clarify what follow-up, if any, was expected and the percentage of patients for which incomplete data or follow-up was obtained
Arians N2019	+	+	+	+	+	+	+	+	?	?	+
Barlow2020	+	+	+	+	+	+	+	+	+	−	+
Immaculada Alonso2011	+	+	+	+	+	+	+	+	+	−	+
Jacek J. Sznurkowski2016	+	+	+	+	+	+	+	+	+	−	+
Kim E. Kortekaas2020	+	+	+	+	+	+	+	+	+	+	+
Larissa J. Lee2016	+	+	+	+	+	+	+	+	+	−	+
Lily Proctor2019	+	+	+	+	+	+	+	+	+	?	+
Linn Woelber2022	+	+	+	+	+	+	+	−	+	−	+
M.L. Yap2018	+	+	+	+	+	+	+	+	+	?	+
Michael J. Dohopolski2019	+	+	+	+	+	+	+	+	+	+	+
Youngkyong Kim2015	+	+	+	+	+	+	+	+	+	−	+
Z.D. Horne 2018	+	+	+	+	+	+	+	−	+	?	+

Risk of bias summary
